# Artificial Intelligence and the Future of Primary Care: Exploratory Qualitative Study of UK General Practitioners’ Views

**DOI:** 10.2196/12802

**Published:** 2019-03-20

**Authors:** Charlotte Blease, Ted J Kaptchuk, Michael H Bernstein, Kenneth D Mandl, John D Halamka, Catherine M DesRoches

**Affiliations:** 1 General Medicine and Primary Care Beth Israel Deaconess Medical Center Harvard Medical School Boston, MA United States; 2 School of Psychology University College Dublin Dublin Ireland; 3 School of Public Health Brown University Providence, RI United States; 4 Computational Health Informatics Program Boston Children's Hospital Harvard Medical School Boston, MA United States; 5 Department of Biomedical Informatics Harvard Medical School Boston, MA United States; 6 Department of Pediatrics Harvard Medical School Boston, MA United States; 7 Beth Israel Deaconess Medical Center Boston, MA United States; 8 Brigham and Women's Hospital Boston, MA United States; 9 Open Notes Beth Israel Deaconess Medical Center Harvard Medical School Boston, MA United States

**Keywords:** artificial intelligence, attitudes, future, general practice, machine learning, opinions, primary care, qualitative research, technology

## Abstract

**Background:**

The potential for machine learning to disrupt the medical profession is the subject of ongoing debate within biomedical informatics and related fields.

**Objective:**

This study aimed to explore general practitioners’ (GPs’) opinions about the potential impact of future technology on key tasks in primary care.

**Methods:**

In June 2018, we conducted a Web-based survey of 720 UK GPs’ opinions about the likelihood of future technology to fully replace GPs in performing 6 key primary care tasks, and, if respondents considered replacement for a particular task likely, to estimate how soon the technological capacity might emerge. This study involved qualitative descriptive analysis of written responses (“comments”) to an open-ended question in the survey.

**Results:**

Comments were classified into 3 major categories in relation to primary care: (1) limitations of future technology, (2) potential benefits of future technology, and (3) social and ethical concerns. Perceived limitations included the beliefs that communication and empathy are exclusively human competencies; many GPs also considered clinical reasoning and the ability to provide value-based care as necessitating physicians’ judgments. Perceived benefits of technology included expectations about improved efficiencies, in particular with respect to the reduction of administrative burdens on physicians. Social and ethical concerns encompassed multiple, divergent themes including the need to train more doctors to overcome workforce shortfalls and misgivings about the acceptability of future technology to patients. However, some GPs believed that the failure to adopt technological innovations could incur harms to both patients and physicians.

**Conclusions:**

This study presents timely information on physicians’ views about the scope of artificial intelligence (AI) in primary care. Overwhelmingly, GPs considered the potential of AI to be limited. These views differ from the predictions of biomedical informaticians. More extensive, stand-alone qualitative work would provide a more in-depth understanding of GPs’ views.

## Introduction

### Background

Although debate about the future of medicine persists, much of the discussion still focuses on recurrent themes such as how health care is paid for and organizational management. In recent years, however, researchers working in the fields of artificial intelligence (AI) and biomedical informatics have begun to raise questions about the potential impact of technology on the medical workforce [1-3]. Although a minority of experts in these fields remain more skeptical that health care is on the cusp of a technological revolution [4], the overwhelming majority of informaticians predict that big data, machine learning, and innovations in AI are poised to significantly overhaul the delivery of medicine [5]. The consensus among these researchers is that core functions of medical professionals—including, but not limited to patient monitoring, diagnostics, and prognostics—will be transformed by technology [6-8].

How might these innovations affect the medical professions? To date, many informaticians forecast that the work carried out by radiologists and anatomical pathologists is likely to be outsourced to algorithms and moreover, that, “The time scale for these disruptions is years, not decades” [9]. When it comes to primary care, however, the predictions appear to be less clear-cut. Many AI researchers suggest that future technology will augment current work practices and eliminate the need for many routine patient visits, but in complex clinical cases, physicians will still be needed to coordinate care and provide empathic support to patients [10-12]. Other AI experts hint at a stronger forecast, suggesting that, in the long term, primary care is vulnerable to disintermediation with physicians eventually being replaced by machine learning algorithms and paraprofessionals [3,8,13].

In opposition to these views are the perspectives of primary care physicians, some of whom claim that the threat of AI is exaggerated. For example, Verghese and colleagues forcefully argue that technological innovation will not significantly encroach on general medicine and “concerns about physician ‘unemployment’ and ‘de-skilling’ are overblown” [14]. They argue that, even if computers provide more accurate diagnoses and prognoses than humans, physicians’ clinical judgments will still be necessary in decision-making processes—as will their expertise in explaining medical information to patients and in the provision of care [14]. Even more emphatically, others have argued that the medical community must guard against, what are perceived to be, the damaging effects of current (and future) technology in general medicine [15]; the use of technology, it is claimed, threatens the quality of patient-centered care which, according to these commentators, necessitates a dyadic, face-to-face interaction between physicians and patients [16,17].

### Objectives

Amid the debate and uncertainties surrounding the impact of AI on the future of the medical professions, we observe that limited attention has so far been paid to the views of practicing physicians [18-22]. Addressing this research gap, we employed quantitative methods to investigate UK-based general practitioners’ (GPs’) opinions about the potential impact of future technologies on primary care. Given the potential for finer-grained insights to be acquired using qualitative methods, we incorporated a single open-ended question into the survey. Our aim was to provide a preliminary investigation into GPs’ perspectives on the themes of the quantitative survey: namely, the bearing of technological innovations on the future of their profession. To our knowledge, this is the first such exploratory investigation of GPs’ opinions and attitudes about AI and the future of a medical specialism.

## Methods

### Main Survey

A complete description of the survey methods and quantitative results has been published previously [22]. In summary, we conducted an anonymous nationwide Web-based survey of UK GPs (response rate=48.84% [720/1474]). All procedures were approved by the ethics review board of University College Dublin; in addition, ethical exemption was approved by the institutional review board of Beth Israel Deaconess Medical Center, Harvard Medical School.

Participants were randomly sampled from membership of the clinician marketing service Doctors.net.uk [23]. This is the largest online medical network in the United Kingdom, and 86.95% (53,670/61,724) of registered and licensed UK GPs are members. We predicted a response rate of around 46% based on previous surveys using this platform [24-26]. Invitations were emailed and displayed on the Doctors.net.uk home pages of 1474 randomly selected GPs for 1 week (June 12-18, 2018). The sample was stratified according to gender and age using up-to-date demographic information about UK GPs provided by the General Medical Council [27]. Invited participants were advised that their identity would not be disclosed to the research team, and all respondents gave informed consent before participating. On completion, respondents were recompensed for the time with £10 (US $13, €11) worth of credit exchangeable for online shopping.

The study team devised an original survey instrument specifically designed to investigate GPs’ opinions about the impact of future technologies on primary care (see Multimedia Appendix 1). We avoided terms such as “algorithms” and “machine learning” in favor of generic descriptors such as “machines” and “future technology.” This was in part to avoid any confusion among physicians unfamiliar with this terminology and to avert technical debates about the explanatory adequacy of specific AI terms of art. The survey was developed in consultation with UK-based GPs (n=6) and primary care physicians in the United States (n=6) to ensure face validity.

### Qualitative Component

To maximize response rate for the qualitative component, the survey instrument included a single open-ended question that allowed participants to respond in more detail on the topic of the questionnaire. Specifically, respondents were asked to “...provide any comments on the survey topic.” Descriptive qualitative data analysis was used to investigate these responses [28,29].

We carried out inductive thematic coding of the data; in light of the limitations of the dataset, a full thematic analysis was not applicable [30]. Responses were collated and imported into QCAmap (coUnity Software Development GmbH) for analysis. The comment transcripts were initially read numerous times by CB to achieve familiarization with the participant responses. Afterward, an inductive coding process was employed in which brief descriptive labels (“codes”) were applied to each comment. Multiple codes were applied to comments with multiple meanings. Comments and codes were reviewed and compared with investigate similarities and differences. CB and TJK met to discuss coding decisions, and subsequent revisions were made. First-order codes were grouped into second-order categories based on the commonality of their meaning to provide a descriptive summary of the responses.

## Results

### Overview

The survey had an overall response rate of 48.84% (of the 1474 GPs invited to participate, 720 responded) [22]. As outlined in the published quantitative survey, respondents were representative of UK GPs in terms of age and gender and from all regions of the United Kingdom [22]. A total of 9.1% of respondents (66/720) left comments (2096 words), which were typically brief (1 phrase or 1 or 2 sentences). GPs who submitted comments were not significantly different from those who did not both in terms of gender and whether they worked part time; however, older GPs (aged 45 years and older) were more likely to leave comments than younger GPs: 83% (55/66) of older GPs left comments, compared with 48.0% (314/654) of older GPs who did not; Table 1). A series of Mann-Whitney *U* tests verified that those who provided qualitative feedback did not differ from those who did not provide qualitative feedback, on the perceived likelihood that future technology would replace human doctors for any of the 6 tasks: *p* *s*>.19.

As a result of the iterative process of thematic analysis, 3 major categories of GPs’ views were identified in relation to primary care: (1) limitations of future technology, (2) potential benefits of future technology, and (3) social and ethical issues. These categories were further subdivided into 8 themes, which are described below with illustrative comments; numbers in parentheses are identifiers that ascribe comments to individual participants.

### Limitations of Future Technology in Primary Care

#### Empathy and Communication

Respondents’ comments encompassed a number of perspectives reflecting perceived limitations of future technology in general practice. One of the main themes identified was the importance of face-to-face, human communication, with the concurrently held belief that technology cannot substitute for humans in providing empathic care. GPs claimed, for example:

It is unlikely that the human element of empathy and the subtlety of human communication and non-verbal cues can be detected by robots or machines.

Participant 517

Technology will never attain a personal relationship with patents. We are essentially a people business. It’s personal relationships that count.

Participant 45

More strongly, some participants argued that physicians would be necessary to provide this care, and moreover, that patients would desire it. For example:

Technology will never be satisfactory as patients are looking for that interaction and dopamine squirt (doctor is the drug) which can only be achieved through empathic continuity of care of highly experienced General Practitioner specialists.

Participant 686

#### Clinical Reasoning

Multiple participants expressed skepticism about the capacity of technology to undertake processes related to clinical reasoning and diagnostic judgments. Some suggested that there was an ineffable aspect to medical decision making that renders it an intrinsically human pursuit:

Technology cannot replace doctors. There is definitely a 6th sense.

Participant 635

Notably, some GPs also implied—either directly or indirectly—that physicians are necessary to gather patient information; a process which, in turn, was deemed indispensable to diagnostics:

The other issue is the inability of a machine/AI to be able to skillfully ascertain the data required from a patient for correct analysis.

Participant 453

**Table 1:** Comparison of general practitioners who did (n=66) and did not (n=654) supply qualitative comments.

Characteristic	Qualitative comments	No qualitative comments	Comparison		
			Chi-square (*df*)	*t* test (*df*)	*P* value
Female (N=720), n (%)	28 (42)	295 (45)	0.2 (1)	Not applicable	.68
Part time, (N=720), n (%)	36 (55)	353 (54)	0.01 (1)	Not applicable	.93
Year of qualification, mean (SD)	1990.1 (9.81)	1996.1 (9.04)	Not applicable	5.15 (718)	<.001
Age≥45 years (N=720), n (%)	55 (83)	314 (48)	29.9 (1)	Not applicable	<.001

They [patients] are humans and subject to the vagaries of human recall, memory, and interpretation. AI may make it easier to interpret a blood result follow a protocol or order a test. But AI will always struggle when the same human can score 1/10 for a symptom today and 10/10 tomorrow.

Participant 201

#### Patient-Centeredness

Comments expressed varying degrees of cynicism about how technology might provide care that is respectful of, and responsive to, individual patient preferences, needs, and values. Some participants considered it improbable that AI could oversee shared decision making, deeming these aspects of patient care to require human competencies. For example:

Medicine, particularly general practice, is an art; listening to ideas concerns and expectations and negotiating a shared plan with the patient. Often doing nothing other than listening is required. I wonder how well a computer will be able to do this?

Participant 285

Technology won’t replace GPs as patient management is about negotiation and managing risks and different patients have different views.

Participant 703

In summary: many participants appeared unpersuaded about the potential for technology to overhaul general practice. This was supported by comments that accentuated the intricacies of the work carried out by physicians. For example:

So much of ill health is vague, complicated and psychological, and the lack of IT to any time soon pick up on individualized, non-verbal cues etc. I feel will still leave a huge role for people.

Participant 457

### Benefits of Future Technology in Primary Care

Although comments encompassed perceived benefits of AI advancements in primary care, notably these were frequently couched in wider perspectives about the predicted shortcomings of future technology. In the main, opinions suggested that such innovations would be restricted to improving performance within traditional GP roles.

#### Improved Efficiencies

A broad consideration was that future technology would improve GPs’ productivity and workflow efficiencies. Some individuals expressed this viewpoint forcefully:

Please hurry up with the technological advances to take away some of the crap that I still have to sort out–then I will be able to get back to proper diagnosing and doctoring.

Participant 693

In this way, some GPs identified a positive paraprofessional role for technology in streamlining access to physicians:

Machines should be good at initial triage of uncomplicated patients presenting to primary care.

Participant 88

Supporting this perspective, some respondents were adamant that advancements in AI would buttress rather than replace the core roles of GPs and “help GPs with workload issues” [Participant 517].

#### An Administrative Role

Extending this viewpoint, many GPs expressed the idea that AI would reduce the burdens of paperwork and provide clerical assistance. For example:

Be useful to develop AI to do analyses of pathology returns, and read all the letters, to provide another presence in the consulting room, and to write the referral letters, organize investigations and the like, ie, act like a personal assistant might do.

Participant 135

I think technology’s place is more about informing patients about conditions and management booking appointments ordering prescriptions contacting the surgery via the internet rather than the phone.

Participant 683

The dominant viewpoint was that technology will reduce the onuses of form-filling and provide administrative support.

### Social and Ethical Concerns in Present and Future Primary Care

#### Understaffing

Interestingly, many participants chose not to interpret the question as directly asking about the impact of AI on the future of primary care, and instead, commented on the growing pressures on the GP workforce, including the risks that this was believed to pose to professionals and patients:

The only reason that I'm not burned out is that I reduced my workload and traded money for sanity.

Participant 280

I changed job due to stress as where I worked I had an unsafe workload

Participant 608

I hate the current stress due to understaffing which is so dangerous

Participant 516

Perhaps, consistent with the prevalent viewpoint that technology would play a limited role in primary care, some respondents stated that increased recruitment of physicians would relieve current demands on the GP workforce:

General Practice in the U.K. is teetering on the brink of collapse due to years of under investment in training adequate numbers of doctors to deal with an ageing population.

Participant 331

Not enough is done to retain experienced GPs. Would save NHS a fortune.

Participant 635

#### Acceptability of Artificial Intelligence

The social implications for patients of possible advancements in AI received much less attention: comments instead were focused on whether the public would be satisfied with, or open to new technology or different ways of obtaining primary care:

The question is whether they will be acceptable to patients although they may be very accessible compared to the current system.

Participant 88

The somewhat blunt tool of technology as it stands will need to evolve some way before the culture of clinicians and patients will accept it.

Participant 453

#### The Ethics of Innovation

Some GPs conveyed greater certainty that AI would lead to major disruptions within general practice though they were unspecific about the nature of these disruptions; for example:

Medicine will be unrecognizable compared to its present form in 25 years.

Participant 282

In respect of this, concerns about safety and accountability were touched on but not described in detail; for example:

Technology will be supporting clinicians in the very near future – the issue is responsibility and liability in legal terms for such tools.

Participant 453

Striking, and in opposition to the overriding outlook among participants, a few respondents considered the preservation of current work practices to be a source of present and future harm. For example:

Risk-taking is not admired or valued yet without it – or AI – general practice will be destroyed.

Participant 464

Technology and non-medical clinicians can replace GPs easily. My burnout is because of my frustration with colleagues and their Luddite working practices.

Participant 495

## Discussion

### Principal Findings

This initial qualitative study affords new insights into how UK GPs view the impact of future technology on primary care. The dominant perspective among respondents was one of skepticism—most GPs believed that the scope of technological innovations will be considerably restricted within general practice. Empathy and communication, in particular, were viewed as quintessentially human skills, and some respondents were adamant that patients will always desire physician-mediated care. Other participants considered doctor-patient interaction as necessary to the process of efficient medical information gathering; similarly, clinical acumen was often assumed to be a uniquely human expertise. GPs viewed the provision of patient-centered care as an interpersonal process that is unlikely to be threatened by automation. Reflecting these themes, expected benefits of AI were generally limited to efficiencies within current models of practice and in particular, to reducing administrative burdens.

Weighing in on wider social and ethical concerns, many GPs reported high levels of burnout, stress, and fears about unsafe workloads. Some comments forcefully expressed the view that greater investment in physician manpower is required. Taking a different perspective, other comments predicted that radical change to primary care was imminent with some GPs claiming that embracing technological innovations is an ethical responsibility to reduce workloads and prevent patient harm.

This exploratory survey suggests that GPs and informaticians are far apart in their views about the impact of machine learning in primary care. In contrast with many of the comments in our study, biomedical informaticians forecast that—both in the short- and long-term—the key functions of primary care will be radically transformed by AI [3,5,6,8,13,31,32]. Indeed, evidence challenges the assumption that physicians are necessary to gather health information [33,34]. Mobile health (mHealth) apps already allow patients to track and monitor a growing number of their own signs and symptoms (eg, blood glucose levels, blood pressure, and levels of physical activity) without the need for traditional checkups with their physician. For example, recent research indicates that home monitoring may be preferable for controlling and preventing chronic conditions: evidence from systematic reviews and meta-analyses of patients with type 1 and type 2 diabetes suggests that mHealth provides clear improvement over clinical and nonmobile interventions in glycemic control and patient self-management [33,34]. Similarly, a 10-year multicenter study of home monitoring for high blood pressure found that ambulatory tracking was not only more accurate but also safer than readings conducted in doctors’ offices: the authors concluded that “white-coat hypertension is not benign” and can mask risk of hypertension among patients [35]. In summary: in contrast with GPs, AI health researchers predict that wearable devices with the capacity for real-time monitoring will improve precision in information gathering while also driving down unnecessary appointments and health care costs [36].

GPs also expressed broad cynicism about the prospects for AI undertaking diagnoses. Again, this perspective is diametrically opposed to the views of biomedical informaticians who argue that the accumulation of “big data”—the collection of vast amounts of information about individual patients (from the genomic and molecular levels, to information about diet, lifestyle, and other environmental factors)—when combined with machine learning, will yield more precise patterns about our individual health and medical outcomes and do so more quickly than humans are able to discern [5-7,9-11,35]. According to AI experts, the capacity to extract novel insights from large health scale data is “where machine learning shines,” with the promise to afford discoveries of hitherto undetected subtypes of diseases [9]. Mining this information for regularities and patterns, and applying algorithmic predictions to new data, it is claimed, will lead to unprecedented personalized precision in diagnostics, prognostics, and treatment recommendations [7,9]. Indeed, aside from medical histories and patient reports, an exponentially increasing volume of health-related information generated from social media posts, apps, purchases, and credit card usage is already being used to support predictions about patient behavior and well-being [37]. In short, beyond the intentional use of digital health devices to undertake diagnostic and prognostic assessments, a vast range of nonmedical data are beginning to yield inferences about patient health, thereby challenging the traditional boundaries of medical expertise [35].

Despite these differences in outlook, GPs shared with many informaticians the view that technological advancements are unlikely to substitute physicians in the provision of empathic patient care [9,10]. Many AI experts argue that humanistic care will be improved with developments in machine learning: they suggest that by outsourcing precision clinical decision making to machine learning algorithms, physicians will be set to invest greater time attending to the needs of their patients. On the other hand, not all AI experts are sanguine about the future role of physicians, or indeed of people, in overseeing humanistic aspects of care [13]. For example, some researchers working in the field of affective computing point to findings that computers can already outperform humans when it comes to accurate discernment of facial expressions [38] and judgments about personality [39].

Comments also incorporated assumptions about patients’ preferences. Some GPs’ assumed that patients would prefer to receive medical care from physicians and raised concerns about the acceptability of future technology among health care users. Although there is limited research either to support or negate these claims, one recent US survey found significant differences between consumers and health providers with respect to their views about mobile technologies [19]. Boeldt and colleagues concluded that consumers were more likely to prefer, and to feel comfortable about, the use of technology for diagnoses than health providers [19].

Interestingly, few comments touched on safety issues: compared with GPs studied, AI researchers identify a wide range of serious concerns related to the design and use of machine learning algorithms. These include the risk of unfairness associated with “algorithmic biases,” which can arise when demographic groups are underrepresented in training phases of machine learning [40]; the reliability and validity of medical algorithms [40]; problems of transparency in determining how algorithms reach decisions [41]; the adequate regulation of apps and mobile technologies [42]; and issues related to privacy and security with respect to patients’ sensitive health information [43,44].

Finally, GPs’ comments about levels of burnout raise important questions about how AI might mitigate unsafe workloads. Even if new machine learning technologies aim to augment human clinical decision making, it is unclear whether these tools will alleviate current levels of physician burnout [8,45]. Conceivably, in the short- and medium-term, if such clinical decision support systems are not suitably designed for human clinical workflow, they may result in “alert fatigue,” undermining their utility [45,46]. Therefore, AI applications that aim to augment clinical judgments need to be designed with human and ergonomic factors in mind.

### Strengths and Limitations

The study has a number of limitations. Comments were often brief, and because of the restrictions of online surveys, it was not possible to probe participants’ responses to obtain a richer understanding of their views. Focus groups or in-depth qualitative interviews would have allowed finer-grained analysis of GPs’ opinions. Given the often short, yet diverse range of opinions articulated in this survey, and in light of omissions of key questions about the potential impact of technology on primary care and the professional roles of physicians, further qualitative work is warranted.

Notwithstanding these limitations, this study provides a foundational exploration of physicians’ views about the future of a medical specialism. The themes support the results of the earlier published quantitative survey while providing more nuanced perspectives on GPs’ opinions about the future of primary care. We recommend that additional qualitative research focus on the attitudes of physicians working in other medical specialties as well as the views of nurse practitioners and physician assistants about how AI may encroach on both the future of patient care and the medical workforce. Finally, when it comes to technological advancements, we urge that greater attention be given to the attitudes and opinions of the users of primary care—patients.

### Conclusions

This preliminary descriptive analysis provides insights into the ways in which GPs think about the impact of technological advancements on primary care. Perceived limitations and benefits of technology and social and ethical concerns about the future of general medicine have been elucidated. A variety of opinions were expressed reflecting some divergence in perspectives; overwhelmingly, however, participants were skeptical about the scope of technology to encroach on the traditional role of the GP (ie, in undertaking patient examinations; performing diagnoses; and providing personalized, empathic care) with only a few considering changes to current practices likely. Notably, this outlook stands in opposition to the predictions of biomedical informaticians and experts working in health care AI.

As we consider these findings, we cannot help but reflect on GPs’ contrastive expectations that future technology will procure benefits in reducing administrative tasks. Such views raise questions about the equanimity of participants’ forecasts. Similarly, some GPs were adamant that technology could not replace physicians in the delivery of empathic care. Although many AI experts appear to agree with this outlook, we suggest that this shared viewpoint does not yet future-proof the role of GPs in overseeing this task: conceivably, if informaticians’ predictions are borne out, nurse practitioners, or a new occupation of medical empathizers, may emerge to undertake humanistic tasks. Relatedly, comments that greater investment in primary care physicians could address workloads are challenged by findings of the World Health Organization, which claims that there will be a worldwide shortage of 18 million health care workers by 2030, over twice the current shortfall [47]. Increasing numbers of patients suffering from chronic illness and aging populations have therefore led many commentators to suggest that new strategies will be required to cope with growing national as well as global health care needs [9,48]. Nonetheless, we caution that the issue of physicians being overworked is unlikely to abate easily. As physicians and providers are currently overwhelmed, burnout itself may be a barrier in the implementation of new tools that are aimed at streamlining the medical care problem [45,49].

Although we cannot provide evidence of explanations for GPs’ skepticism, it seems plausible that respondents’ beliefs may reflect a level of disengagement from the literature on health care AI [50]. Our findings carry implications about the capacity of physicians to contribute in meaningful and objective ways to the many cutting-edge ethical and policy issues in relation to the advancement of AI in medicine [8,51,52]. Therefore, we conclude that our survey results raise important questions about the adequacy of medical curricula to equip future physicians for potential changes to clinical practice and, thereby, to lead and shape crucial debates about the future of patient care. Improvements in education, we suggest, may go some way to closing the rift between current AI health researchers and practitioners.

## Appendix

Multimedia Appendix 1 Survey questions.

## Abbreviations

AI: artificial intelligence
GPs: general practitioners
mHealth: mobile health
